# Supplementary material for: Selective inhibition of respiratory complex I reveals a bioenergetic vulnerability in Francisella
Source: bioRxiv. 2026 Jun 5:2026.06.04.730185. Preprint. [Version 1] doi: 10.64898/2026.06.04.730185 (PMC13251983; doi:10.64898/2026.06.04.730185)
Supplement: 1 [file NIHPP2026.06.04.730185v1-supplement-1.pdf]

bioRxiv preprint doi: https://doi.org/10.64898/2026.06.04.730185; this version posted June 5, 2026. The copyright holder for this preprint (which was not certified by peer review) is the author/funder, who has granted bioRxiv a license to display the preprint in perpetuity. It is made available under a CC-BY 4.0 International license.

SUPPLEMENTARY FIGURES

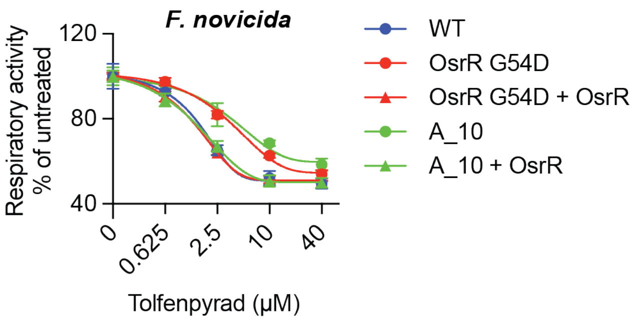

Fig. S1. Tolfenpyrad-resistant *F. novicida* strains exhibit reduced respiratory inhibition. Respiratory activity measured by MTT reduction in wild-type (WT) *F. novicida*, and a tolfenpyrad-resistant mutant and isolate (OsrR G54D and A_10, respectively), as well as the mutants expressing wild-type OsrR following treatment with the indicated concentrations of tolfenpyrad. Data are presented as mean ± S.D. and are representative of three or more independent experiments performed with at least three technical replicates per condition.

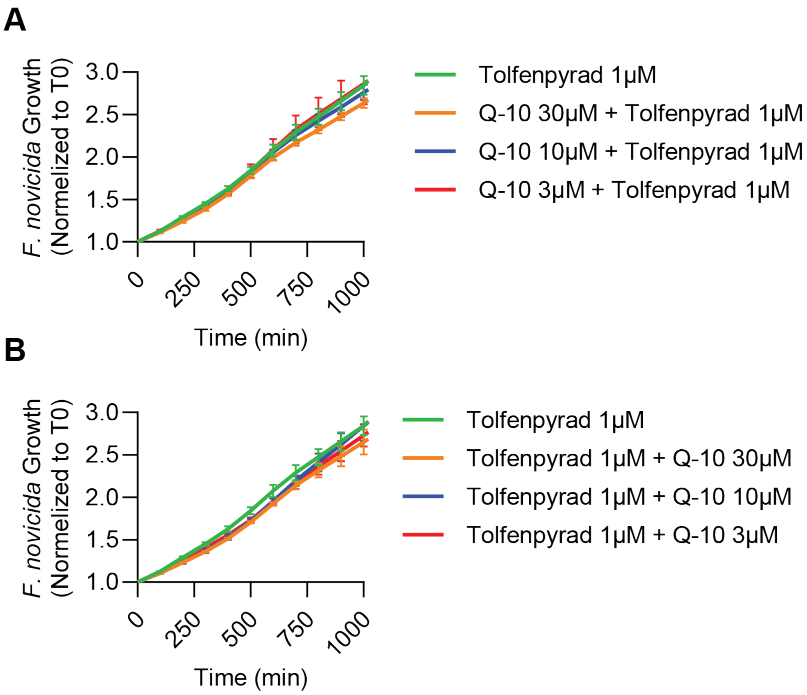

Fig. S2. Exogenous ubiquinone does not rescue tolfenpyrad-mediated growth inhibition. (A) Growth of *F. novicida* following pre-incubation with the indicated concentrations of coenzyme Q10 prior to treatment with 1 µM tolfenpyrad. **(B)** Growth of *F. novicida* following treatment with 1 µM tolfenpyrad before addition of coenzyme Q10.
